# Synthesis and Phase Evolution of a Nanocrystalline Fe_x_CrNiAl (x = 1.0, 0.5, 0.25) High-Entropy Alloys by Mechanical Alloying

**DOI:** 10.3390/ma17246061

**Published:** 2024-12-11

**Authors:** Danni Yang, Mingqing Liao, Jingtao Huang, Tianyi Han, Nan Qu, Yalin Wang, Jingchuan Zhu

**Affiliations:** 1 School of Materials Science and Engineering, Lanzhou University of Technology, Lanzhou 730050, China; 2 State Key Laboratory of Advanced Processing and Recycling of Non-Ferrous Metals, Lanzhou University of Technology, Lanzhou 730050, China; 3 School of Materials Science and Engineering, Jiangsu University of Science and Technology, Zhenjiang 212100, China; liaomq1900127@163.com; 4 Department of Forensic Science and Technology, Zhengzhou Police University, Zhengzhou 450053, China; huangjingtao@rpc.edu.cn; 5 School of Materials Science and Engineering, Harbin Institute of Technology, Harbin 150001, China; hantianyi@hit.edu.cn (T.H.); qunan1967@163.com (N.Q.); 6 Key Laboratory of Special Functional Materials and Structural Design, Ministry of Education, Lanzhou University, Lanzhou 730050, China

**Keywords:** high-entropy alloys, mechanical alloying, nanocrystalline, phase evolution

## Abstract

High-entropy alloys (HEAs) with ultrafine grained and high strength can be prepared by mechanical alloying (MA) followed by sintering. Therefore, MA, as a unique solid powder processing method, has many effects on the microstructures and mechanical properties of the sintered bulk HEAs. This work focused on the alloying behavior, morphology, and phase evolution of Fe*_x_*CrNiAl (x = 1.0, 0.5, 0.25) HEAs by MA. The X-ray diffraction results show that the powders achieved a supersaturated solid solution body-centered-cubic (BCC) phase after MA; the crystalline size reached the nanoscale and was refined to ~80 nm. The morphology and composition of the alloyed powders were studied by scanning electron microscopy with energy dispersive spectroscopy. The results indicate that the powder was decreased to 1.59 μm for Fe_1.0_ powder with excellent homogeneity in composition. There exists a phase transformation during high-temperature annealing, as the non-equilibrium BCC supersaturated solid solution phase transformed into the equilibrium phase of BCC and ordered BCC (B2) phases.

## 1. Introduction

High-entropy alloys (HEAs) have attracted extensive attention since 2004 for their excellent properties [1,2]. Due to the high configuration entropy, the alloy tends to form simple solid solution phases instead of intermetallic compounds, such as BCC, FCC, and HCP [3,4]. In addition, the alloys are composed of multi-principal-component elements, and each component can be regulated in a wide range of atomic percentages. A vast realm of alloy composition regulation makes it possible to achieve comprehensive mechanical properties, such as high hardness and strength [5,6], great oxidation resistance [7,8], and excellent wear and corrosion resistance [9,10], which are in great demand for the industry as loading-bearing structures.

Vacuum arc melting is the primary method of fabricating bulk HEAs [11,12], where all components are fully mixed in the liquid state and then solidified in a copper crucible [11,12]. However, the method is always accompanied by the defect of composition segregation and coarse dendritic microstructures, which are hardly improved in the following treatment, resulting in the degradation of the mechanical properties of HEAs [13]. Therefore, plenty of methods have been developed to enhance the mechanical properties of HEAs, such as powder metallurgy [14,15,16], laser cladding [17], and additive manufacturing [18,19], among which powder metallurgy, as a low-cost and rapid way to prepare HEAs, is an ideal method for overcoming casting defects. For this method, the alloyed powder is usually synthesized by mechanical alloying (MA), and the following high-temperature sintering step can consolidate it to a bulk HEA [14,15,16]. Thus, the performance of the sintered bulk alloy is closely related to the alloyed powder synthesized by the MA process [20].

Mechanical alloying (MA) is a powder processing route to fabricate alloys with elemental powders in the solid state [21]. The non-equilibrium supersaturated phase is obtained by high-energy ball milling with repeated cold welding and fracture [21]. In addition, homogeneous composition and even a nanoscale crystalline size can be easily synthesized by the MA process [22,23,24]. Combined with the following high-temperature sintering, bulk HEAs with ultrafine grains and high strength can be obtained. Fu [16] synthesized nanocrystalline Co_25_Ni_25_Fe_25_Al_7.5_Cu_17.5_ FCC HEA via MA and spark plasma sintering. The yield strength was increased by 834.9%. Jiang [20] sintered the AlCrFeNi MEA by using powders under different status, and the results indicate that the powders have evident effects on the microstructures and mechanical properties of the bulk HEAs. Therefore, it is necessary to study the MA process systematically.

In this work, taking into consideration the lack of Co and the light weight, the FeCrNiAl alloy system was chosen as a model alloy to fabricate the nanocrystalline Fe*_x_*CrNiAl (x = 1.0, 0.5, 0.25) HEAs by MA. The alloying behavior, morphology, and phase evolution of the HEA powders were systematically investigated. High-temperature vacuum annealing was performed to study the high-temperature phase evolution of the alloyed powders.

## 2. Experimental Details

The elemental powders of Fe (99.5 wt.%), Cr (99.5 wt.%), Ni (99.5 wt.%), and Al (99.5 wt.%) were selected as the raw materials, with a particle size less than 45 μm. The alloyed Fe*_x_*CrNiAl (x = 1.0, 0.5, 0.25) powders were synthesized by a high-energy planetary ball machine (QM-3SP4 Planetary Ball Mill; Nanjing Nanda Instrument Plant, Nanjing, China). The mixed powders were milled in stainless steel vials for 40 h at 350 rpm under an Ar atmosphere. The milling media was a WC ball, and the ball/powder ratio was 10:1. The mass ratio of the milling balls with diameter of 10 mm and 5 mm was 13:7. The milling was suspended for 5 min every 30 min. To avoid overheating during MA, the powders were milled by dry milling for 35 h and then by wet milling for 5 h, and the process-controlling agent was 3 wt.% ethanol during the wet milling. The milled powders were taken out in a glovebox filled with Ar. The alloyed powders were annealed in a sealed quartz tube under the vacuum by a muffle furnace with a pressure of 10^−2^ Pa. The annealing treatments were carried out at 800 °C, 900 °C, and 1000 °C for 60 min, respectively.

X-ray diffraction (XRD; Empyrean, Panalytical, Almelo, The Netherlands) with Cu *K*_α_ radiation (*λ* = 0.15419 nm) was used to characterize the phase structure. The microstructures and elemental composition were analyzed by scanning electron microscopy (SEM; Supra55, Zeiss, Oberkochen, Germany) equipped with an energy dispersive spectrometry device (EDS; Oxford Instruments, London, UK). A differential scanning calorimeter (DSC; STA449F3, Netzsch, Selb, Germany) was utilized under the flow of high-purity Ar, and the heating rate was 10 °C/min.

## 3. Results and Discussion

### 3.1. Alloying Behavior and Morphology of the Powders

**Figure 1:**
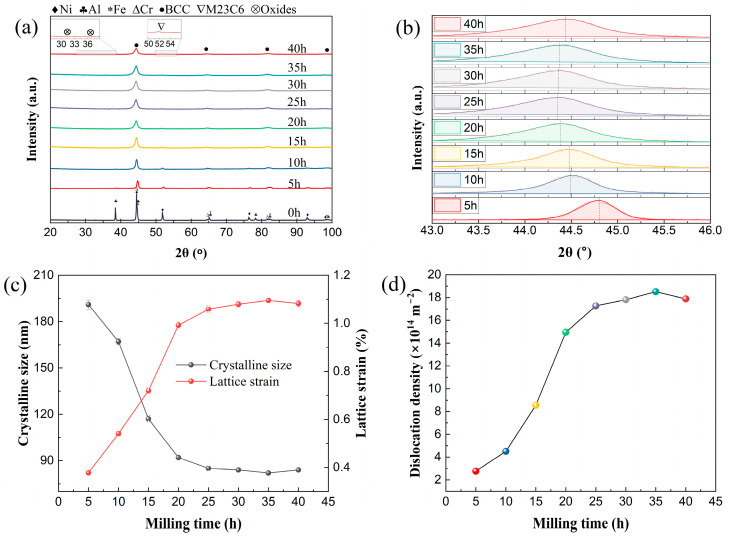
The phase evolution of Fe_1.0_CrNiAl alloyed powders with different milling times: (**a**) and (**b**) XRD patterns and the fitted main peaks; (**c**) the grain size and lattice strain; (**d**) the dislocation density.

Figure 1 characterizes the phase evolution of Fe_1.0_CrNiAl powders at various milling times by the XRD patterns. The pure elemental powders were mixed primitively and marked as 0 h in Figure 1a. All diffraction peak intensities decreased obviously in a short time, with the intensity at 2θ = 38° decreasing rapidly after 5 h of milling. The Al started alloying first, and finished the alloying at 15 h; meanwhile, the dissolution of Ni started, and the broadening of peaks occurred significantly. After milling for 25 h, Ni peaks disappeared to finish the alloying, and the Fe and Cr completed alloying after one another. At 25~35 h, sufficient diffusion and dissolution play a primary role; the peaks broadening hardly changes during this stage, indicating a balance between welding and fracturing during milling. The BCC solid solution phase formed after 40 h of milling, and the weak peaks of the oxides and carbides were also detected due to the introduction of the process-controlling agent [21,25].

The broad peaks were ascribed to the reduction in grain size and the increment of lattice strain during the milling, which was evaluated via the Williamson–Hall method [26] after eliminating the instrument contribution. The equation is $B{\cos\theta }_{B}=\frac{Kd}{\lambda }+\varepsilon {\sin\theta }_{B}$, where *K* is ~0.9, *λ* is the wavelength of a Cu *K*_α_ X-ray (0.15419 nm), and *ε* and *θ_B_* are the lattice strain and the Bragg angle, respectively [26,27]. The crystalline size together with the lattice strain of Fe_1.0_CrNiAl is displayed in Figure 1c. The work hardening increased quickly, resulting in the accumulation of dislocations [21] during 1~15 h, and the crystalline size was refined rapidly from 191 nm to 117 nm in this period. With a continuous increasing in lattice strain, the grain disintegrated into sub-grains at heavily strained regions. During 15~25 h, the crystalline size decreased slowly and refined to 84 nm after 40 h milling. The crystalline size and lattice strain of the Fe*_x_*CrNiAl powders are listed in Table 1. The alloyed powder of Fe_0.25_ reached values of 80 ± 1 nm and 1.1100 ± 0.0128% when finished the alloying process. According to the fitted diffraction peaks in Figure 1b, due to the solution during milling, the main diffraction peaks shift left by 0.31 ° from 0 h to 5 h, and slightly shift left 0.142° from 10 h to 25 h. After that, the thermal effect generated by the high-energy ball milling eliminates the work hardening partially, resulting in the rightward shifting of the diffraction peak [21]. The dislocation density generated during the process was calculated by $\rho =\frac{2\sqrt{3}\varepsilon }{\mathrm{db}}$, where *d*, *b*, and *ε* are crystalline size, lattice strain, and Burgers vector, respectively [28], which are shown in Figure 1d. The dislocation density of Fe_1.0_ was 2.77 × 10^14^ m^−2^ at 5 h, and rapidly increased to 1.50 × 10^15^ m^−2^ at 20 h. From 20 h to 35 h, due to the recovery and recrystallization of the grains, the value increased slightly and reached 1.79 × 10^15^ m^−2^ after 40 h of milling [16]. The dislocation density of the Fe*_x_*CrNiAl (x = 1.0, 0.5, 0.25) alloy powders is listed in Table 1.

The alloying sequence has a close relationship with the structure and melting point of the alloying element [29]. The properties of the elements are shown in Table 2. Al promotes the formation of the BCC phase in the high-entropy alloy system [30], has a lower melting point but a higher intrinsic diffusion coefficient, and completes the alloying first. This notwithstanding, Ni and Al were easy to combine for the lowest mixing enthalpy [31] in Table 3; similarly, Cr and Fe finished the alloying at the final stage due to the more positive mixing enthalpy in the system. Therefore, the alloying sequence of the FeCrNiAl alloy system was Al→Ni→Fe→Cr, which was consistent with the previous research [32,33,34,35]. In addition to the influence of elements, the mechanical energy was partially converted into heat energy to facilitate the solid-state diffusion of elements, which made for quick alloying of the elements.

**Figure 2:**
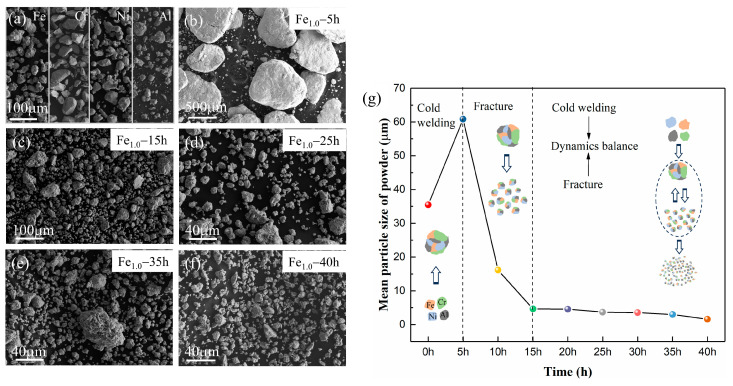
The characterization of Fe_1.0_CrNiAl powder during MA: (**a**–**f**) the micrograph of the powder; (**g**) the mean particle size.

Figure 2 shows the morphology and mean particle size of the milled Fe_1.0_CrNiAl powders at different milling times. The mean particle size of the mixed raw material was statistically about 35 μm. As shown in Figure 2b, at the beginning of MA, cold-welding plays a dominant role; new surfaces were created and enabled the particles to weld together, resulting in larger particles with a wider range of particle size distribution, and the majority of particles were ten times larger than the raw materials. Due to the collision force of the milling balls, the morphology of the particle was polygon layered structured fragments, and the particle size increased rapidly to 60.85 μm after 5 h milling. With the continued milling of 5~15 h, the fracture tendency predominates over cold-welding, the particle experienced severe plastic deformation, and the size gradually decreased to 4.66 μm at 15 h. After 20 h, the particle size was decreased slowly. At this time, a steady-state equilibrium is attained due to the balance between cold welding and fracture; the mean size was 1.59 μm at 40 h. As displayed in Figure 2f, the powder particle tended to be spherically shaped.

**Figure 3:**
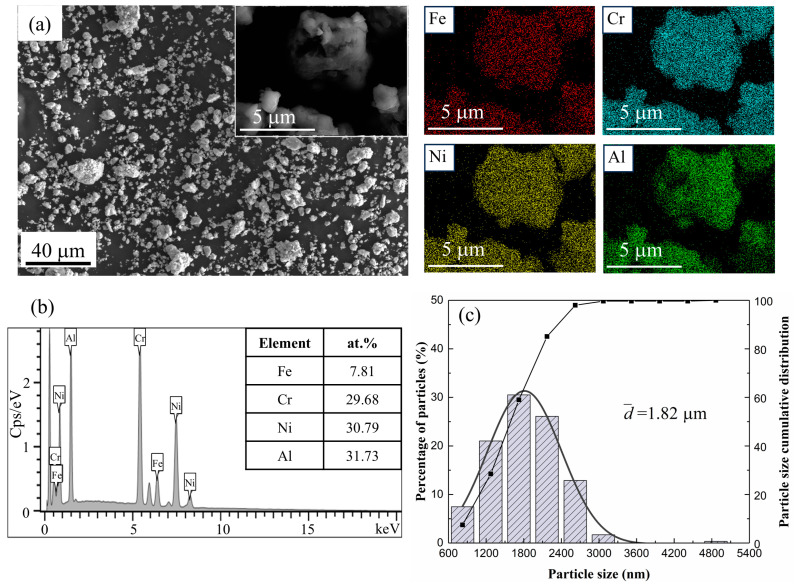
The morphology of the alloyed Fe_0.25_CrNiAl powder: (**a**) the SEM image and the EDS mapping; (**b**) the elemental composition from the mapping; (**c**) particle size distribution.

Figure 3 shows the morphology and EDS results of the alloyed Fe_0.25_CrNiAl powder. The particles displayed a homogeneous elemental distribution with a nearly spherical shape. The EDS spectrum in Figure 3b is presented in tabular form. The results show that uniform alloyed HEA powders can be obtained by high-energy MA. Figure 3c shows the particle size histograms of the alloyed Fe*_x_*CrNiAl (x= 1.0, 0.5, 0.25) powders; as shown in Table 4, the particle size was refined to 1.59 μm, 1.80 μm, and 1.82 μm, respectively, when the mechanical alloying was finished.

### 3.2. Phase Formation and High-Temperature Phase Evolution of the HEA Powders

Various empirical criteria and thermodynamics parameters have been proposed to discuss the solid solution phase formation of the HEAs, such as the mixing entropy ($∆S_{\mathrm{mix}}$), the mixing enthalpy ($∆H_{\mathrm{mix}}$), the Ω-parameter of $\Omega =\frac{T_{m}\Delta S_{\mathrm{mix}}}{\left|\Delta H_{\mathrm{mix}}\right|}$ (*T*_m_ is the mole-averaged melting point of the alloy) [37], the difference in atomic size (*δ*) [38], the valence electron concentration (*VEC*) [39], and the electronegativity difference Δ*χ* [40]. It is worthwhile discussing the solid solution phase formation of Fe*_x_*CrNiAl (x = 1.0, 0.5, 0.25); the calculated values of Fe*_x_*CrNiAl HEAs for $∆S_{\mathrm{mix}}$, $∆H_{\mathrm{mix}}$, Ω, *δ*, *VEC*, and Δ*χ* are listed in Table 5. The values of $∆H_{\mathrm{mix}}$ and *δ* satisfy the condition of 0 ≤ *δ* ≤ 8.5% and −22 ≤ $∆H_{\mathrm{mix}}$ ≤ 7 kJ/mol to form the solid solution phase. The *VEC* is less than 6.87, indicating a simple BCC phase exists in the Fe*_x_*CrNiAl high-entropy alloy system, which is well in agreement with the experimental results [38,40,41,42,43]. Particularly, the $∆S_{\mathrm{mix}}$ of Fe_0.25_CrNiAl is slightly lower than the 11 ≤ $∆S_{\mathrm{mix}}\;$≤ 19.5 J/(K·mol) for its non-equiatomic alloy composition, but Ω > 1 because the contribution of $T∆S_{\mathrm{mix}}$ is over that of $∆H_{\mathrm{mix}},$ resulting in the formation of a mainly solid solution phase [12,44].

**Figure 4:**
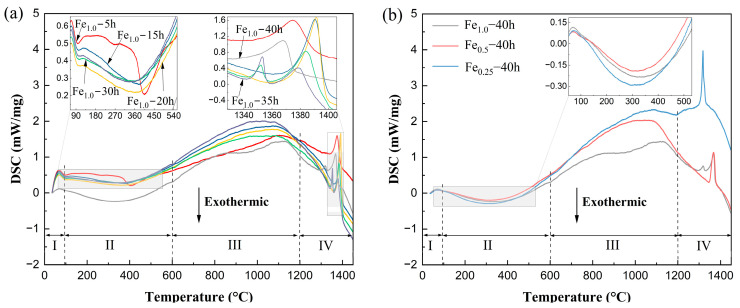
DSC curves of powders: (**a**) the curves of Fe_1.0_ powders during milling; (**b**) the curves of 40 h alloyed Fe_x_CrNiAl (x = 1.0, 0.5, 0.25) powders.

Figure 4 shows the DSC results of Fe*_x_*CrNiAl (x = 1.0, 0.5, 0.25) powders during milling; the endothermic and exothermic peaks have a similar trend for all the powders, and the powders experienced four stages during the heating. The first stage was from room temperature to 100 °C, and the obvious endothermic peaks were mainly associated with the introduction of the process-controlling agent [45,46]. This stage corresponds with the evaporation of the water and ethanol. The second stage was from 100 °C to 600 °C, and the trend was related to the release of internal stresses caused by plastic deformation and lattice strain, etc. [45]. In the third stage, the work hardening caused by plastic deformation was eliminated, the powders started to absorb heat, and the supersaturated solid solution phase began to transform into equilibrium-state phases of BCC and ordered BCC (B2) phases. With continued heating to the fourth stage, the temperature was close to the melting point, and the powder started to absorb heat, leading to the collapse of the crystalline structure. The melting point of the powders was the endothermic peak at 1300~1400 °C. It is worthwhile to note that the powder milled 40 h absorbed less heat compared with that of the 5~35 h milled powders due to the energy stored during MA.

**Figure 5:**
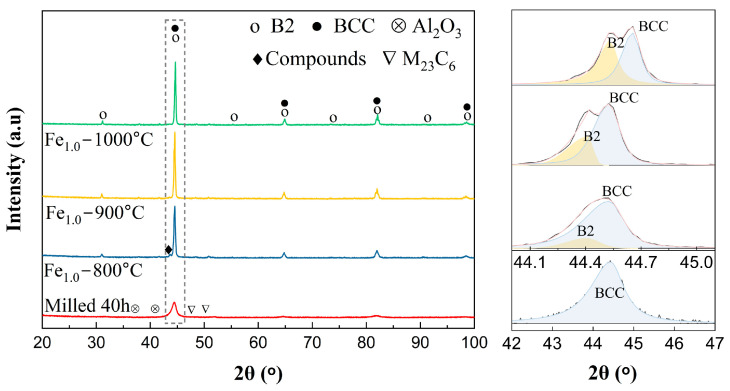
The XRD patterns of Fe_1.0_CrNiAl powder vacuum-annealed under different temperatures.

The alloyed powders were annealed at the temperature of 800~1000 °C, and what followed was to investigate the phase evolution of the alloyed powders. The XRD patterns are shown in Figure 5. The diffraction peaks of the alloyed powders split into two peaks, indicating that the non-equilibrium supersaturated solid solution phase transformed into the equilibrium phase with BCC and ordered BCC (B2) phases after the annealing, and the intermetallic compounds came into being when the temperature was 800 °C. According to the fitted main diffraction peaks of the Fe_1.0_CrNiAl powder, it is clear that the volume fraction of the B2 phase increased with the increasing annealing temperature. The volume fraction of the B2 phase was 11.89% when annealed at 800 °C, while the fraction increased to 26.85% at 900 °C and about 49.44% at 1000 °C. In addition to the phase evolution at high temperatures, it is worth noting that the oxides and carbides formed during the MA process still exist at a high temperature of 1000 °C, which plays an important role in the following sintering process by inhibiting grain growth [8,25].

## 4. Conclusions

The nanocrystalline Fe*_x_*CrNiAl (x = 1.0, 0.5, 0.25) high-entropy alloy powders with supersaturated BCC phases were synthesized by mechanical alloying successfully. The alloying behavior, morphology, and phase evolution of the powders were discussed systematically. The continuous collision of high-energy milling facilitated the diffusion and dissolution of each component; the alloyed powders have a crystalline size of ~80 nm, resulting in a high dislocation density of ~10^15^ m^−2^, which lowered the subsequent sintering temperature. And the powders particles have excellent homogeneity in composition with a mean particle size of 1.59~1.82 μm. The non-equilibrium supersaturated solid solution phase transformed into the equilibrium phase of BCC and ordered BCC (B2) phases after annealing at temperatures from 800 °C to 1000 °C, and the volume fraction of the B2 phase increased with the annealing temperature, reaching about 49.44% at 1000 °C.

## Figures and Tables

**Table 1:** Crystalline size and lattice strain of the Fe*_x_*CrNiAl (x = 1.0, 0.5, 0.25) alloys during MA.

Alloys	Milling Time (h)	Crystalline Size (nm)	Lattice Strain (%)	Dislocation Density (m^−2^)
Fe_1.0_	35	82 ± 1	1.0950 ± 0.0077	1.85 × 10^15^
	40	84 ± 1	1.0820 ± 0.0133	1.79 × 10^15^
Fe_0.5_	35	83 ± 1	1.0860 ± 0.0163	1.82 × 10^15^
	40	81 ± 1	1.0200 ± 0.0107	1.75 × 10^15^
Fe_0.25_	35	81 ± 1	1.1160 ± 0.0081	1.91 × 10^15^
	40	80 ± 1	1.1100 ± 0.0128	1.92 × 10^15^

**Table 2:** The properties of elements included in FeCrNiAl-based alloys [29].

Element	*T*_m_ (°C)	*C.S.* (400K)	*r* (Å)	*D*_0_ (400K)
Fe	1538	BCC	1.27	10^−31^
Cr	1857	BCC	1.28	10^−41^
Ni	1453	FCC	1.25	10^−37^
Al	660	FCC	1.43	10^−19^

*T*_m_: melting point; *C.S*: crystal structure; *r*: atomic size; *D*_0_: self-diffusion coefficient.

**Table 3:** The value of $∆H_{\mathrm{ij}}^{\mathrm{mix}}$ (KJ/mol) for binary equi-atomic alloys calculated by Miedema’s model [36].

Element	Fe	Cr	Ni	Al
Fe	—	−1	−2	−11
Cr	−1	—	−7	−10
Ni	−2	−7	—	−22
Al	−11	−10	−22	—

**Table 4:** The mean particle size and SEM-EDS elemental distribution of Fe*_x_*CrNiAl (x = 1.0, 0.5, 0.25) powders after MA.

Alloys.	Mean Particle Size (μm)	Regions	Fe (at.%)	Cr (at.%)	Ni (at.%)	Al (at.%)
Fe_1.0_	1.59	Nominal composition	25.00	25.00	25.00	25.00
		EDS mapping	26.22	23.56	24.10	26.12
Fe_0.5_	1.80	Nominal composition	14.28	28.57	28.57	28.57
		EDS mapping	15.39	29.26	29.84	25.51
Fe_0.25_	1.82	Nominal composition	7.69	30.77	30.77	30.77
		EDS mapping	7.81	29.68	30.79	31.73

**Table 5:** The values of Fe*_x_*CrNiAl (x = 1.0, 0.5, 0.25) HEAs for $∆S_{\mathrm{mix}}$, $∆H_{\mathrm{mix}}$, Ω, *δ*, *VEC*, and Δ*χ*.

Alloy	Δ*H*_mix_ (kJ/mol)	Δ*S*_mix_ (J/K·mol)	Δ*χ*	*δ* (%)	*VEC*	Ω
Fe_1.0_	−13.2500	11.5256	0.1221	6.2600	6.7500	1.4353
Fe_0.5_	−15.0204	11.2387	0.1268	6.4630	6.5714	1.2174
Fe_0.25_	−16.0947	10.6859	0.1291	6.5587	6.4615	1.0709

## Data Availability

The original contributions presented in the study are included in the article, further inquiries can be directed to the corresponding authors.
